# Effort or ease: the impact of sharing self-improvement vs. hedonic behaviors on personal brand evaluation

**DOI:** 10.3389/fpsyg.2025.1666105

**Published:** 2025-10-20

**Authors:** Chenhan Ruan, Xiaoyang Zhang, Fenglian Zhuo, Zhihuang Lu, Juan Li

**Affiliations:** ^1^School of Economics and Management, Fujian Agriculture and Forestry University, Fuzhou, China; ^2^School of Management, Shanghai University, Shanghai, China

**Keywords:** hedonic behaviors, self-improvement behaviors, personal brand evaluation, intrinsic motivation, social mobility, perceived similarity

## Abstract

Daily activities, as ubiquitous and relatable aspects of human life, have become a pivotal resource for social media influencers to build personal brands, with two primary types identified: hedonic behaviors (e.g., eating dessert) that pursue immediate sensory pleasure and emotional wellbeing, and self-improvement behaviors (e.g., learning) that focus on personal development for eudaimonic wellbeing. While previous studies mainly examined factors influencing consumers’ preferences between these two options, few have explored how such posts serve as signaling cues to shape consumer inferences. Drawing on Social Identity Theory, this study thus aims to investigate the impact of sharing hedonic versus self-improvement posts on personal brand evaluation. Employing four experiments, we tested our hypotheses across different samples and scenarios. The results show that self-improvement (vs. hedonic) posts elicit more positive inferences about sharers’ intrinsic motivation (Study 1), which in turn promotes personal brand evaluation (Studies 2–4). Moreover, two boundary conditions moderate this positive effect: it is attenuated in contexts with low social mobility (Study 3) or among viewers who perceive high similarity with the bloggers (Study 4). Overall, the findings enrich the theoretical understanding of social identification in posted activities, and provide practical implications for personal brand promotion.

## 1 Introduction

With the development of information network, the size of social network market has rapidly increased, along with a growing number of social media users. According to CNNIC data, in January 2025, the number of netizens in China reached 1.108 billion (CNNIC, 2025). People enjoy posting daily life activities on social media platforms, connecting viewers as a real person rather than a distant creator. Such posts not only establish strong authenticity and relatability for personal brands, but also strengthen parasocial bonds with the audience to create a sense of involvement in the bloggers’ life (Elgammal and Majeed, 2024; Yang et al., 2021). When sharing life experiences, bloggers face a fundamental choice between two common types of activities: hedonic activities (e.g., leisure and eating dessert) to show their immediate pleasure, or self-improvement activities (e.g., enhancements in skills and health) to demonstrate their long-term growth. Hedonic behavior is defined as the activity that pursues immediate sensory pleasure and emotional wellbeing, whereas self-improvement behavior refers to activity that seeks personal development and eudaimonic wellbeing (Orgilés et al., 2021; Zhang et al., 2024). Their prevalence stems from the fundamental human pursuit of wellbeing (Ryan and Deci, 2001; Zhang et al., 2024). This raises a critical yet unexplored research question: Which activity yields better personal brand evaluation?

The extant literature has documented insufficient views on this question. Prior studies have mainly focused on how external stimuli such as guilt, romantic experience, and god salience influence consumers’ self-improvement versus hedonic consumption (Allard and White, 2015; Grewal et al., 2022; Zhang et al., 2024), but they failed to explore how sharing engagements with hedonic and self-improvement contents shape consumers’ identifications with the influencers. Moreover, extant research on hedonia – eudaimonia distinction has predominantly focused on how these two types of motivational orientation toward happiness shape individuals’ life goals, motivations, and behavioral responses (Disabato et al., 2016; Steger et al., 2008), but it fails to understand how they influence viewers’ social identification process with sharers. Due to the asymmetry of information between content creators and audiences, shared contents serve as cues for consumers to decode the underlying motives and form impressions toward the personal brands (Kim and Drumwright, 2016; Yang et al., 2021). The lack of insights from viewers’ perspective underscores the need for a systematic investigation into the differential impacts of hedonic versus self-improvement posts on personal brand evaluations.

In this research, we propose a superiority of self-improvement activities over hedonic ones on personal brand evaluation. According to Social Identity Theory (Tajfel and Turner, 1986), self-improvement as socially desirable attributes facilitates group identification and strengthens community associations (Algesheimer et al., 2005; Hughes and Ahearne, 2010). When viewers perceive that bloggers’ self-improvement posts reflect intrinsic motivation (e.g., pursuing satisfaction from challenging tasks, finding the work itself enjoyable), they will be more likely to identify bloggers as authentic ideal group (Lee and Pounders, 2019; Ryan and Deci, 2001). By contrast, hedonic behaviors emphasize immediate sensory enjoyment and instant gratification over long-term meaning. These actions are more likely to be viewed as driven by external temptations (rather than intrinsic motivations), which in turn reduces personal brand evaluations (Disabato et al., 2016; Ryan and Deci, 2001). Moreover, we propose that the social desirability of self-improvement could be influenced by societal and interpersonal factors. Specifically, we propose the superiority of self-improvement (vs. hedonic) activities would be attenuated in societies with lower level of social mobility, or among viewers with high similarity with sharers.

Based on four experimental studies, our research contributes to both personal brand marketing and practice. Firstly, this study enriches the impression formation literature by identifying these two types of behaviors (i.e., hedonic and self-improvement behaviors) as unique antecedents in shaping personal brand evaluations. Secondly, we further reveal that perceived intrinsic motivation mediates the above relationships, showing how the underlying motivational inferences of the posted contents influence the social identification with the bloggers. Thirdly, this article explores the variation of this effect with societal and interpersonal factors rather than predominantly individual factors. From a managerial perspective, this research provides actional suggestions for bloggers to select suitable information and strategically tailor their contents on social media platforms.

## 2 Theoretical background and hypotheses

### 2.1 Effect of sharing hedonic and self-improvement behaviors on personal brand evaluation

A blogger’ s posts on social media can significantly shape consumers’ evaluations (Cheung et al., 2022; Shoukat et al., 2023). Among the behaviors commonly shared, hedonic and self-improvement activities are particularly salient, as they represent fundamental pursuit to wellbeing (Ryan and Deci, 2001). The theory of subjective wellbeing distinguishes these two motivation orientations behind happiness pursuit: one centered on long-term, meaning-based eudaimonia, and the other on immediate, emotion-based hedonism (Disabato et al., 2016; Steger et al., 2008). Hedonic behaviors are characterized by the pursuit of immediate gratification and sensory pleasure (Elgammal et al., 2022; Zhang et al., 2024). People engage in such activities for immediate gratification and enjoyment to achieve hedonic wellbeing (Orgilés et al., 2021). In contrast, self-improvement behaviors reflect individuals’ deliberate efforts to achieve eudaimonic wellbeing through personal growth and the pursuit of long-term goals (Dong et al., 2021).

According to Social Identity Theory, individuals categorize themselves through comparisons with in-group and out-group members, and actively align with those who initiate socially approved behaviors (Algesheimer et al., 2005; Tajfel and Turner, 1986). Self-improvement behaviors are inherently tied to eudaimonic motivational orientations (Steger et al., 2008). They embody socially valued attributes such as disciplined, strong-willed, aspirations toward an ideal self, and signal determination to overcome personal limitations in pursuit of long-term goals (Dong et al., 2021; Du et al., 2025; Grewal et al., 2022). More importantly, when bloggers share self-improvement activities, audiences tend to infer that these actions stem from their intrinsic motivation (e.g., seeking satisfaction from challenging tasks, deriving pleasure from learning; Derfler-Rozin and Pitesa, 2020) rather than external incentives like praise or rewards. The inference of intrinsic motivation strengthens the perception that the blogger is authentic and consistent with the aspirational social identity that audiences aspire to belong to (Grewal et al., 2022). This in turn fosters more favorable evaluations and stronger affiliative intentions (Du et al., 2025).

However, hedonic behaviors such as indulgent consumption, passive leisure, or pleasure seeking, are inherently linked to a preference for instant gratification over long-term self-development (Hu and Min, 2022). Unlike self-improvement behaviors, hedonic actions are more often perceived as reactive responses to external temptations rather than adherence to individuals’ internal values (Hollebeek et al., 2023). Besides, this distinction becomes particularly relevant among bloggers, since actions that support personal growth are often seen as markers of the aspirational social identity valued in mainstream context. When bloggers share hedonic behaviors, they may appear less committed to personal growth or the pursuit of an ideal self, thereby diminishing their perceived social value (Grewal et al., 2022). Based on the previous discussion, we propose that:

*H1: Compared to sharing hedonic behaviors, sharing self-improvement behaviors leads to higher personal brand evaluation.*

### 2.2 The mediating role of perceived intrinsic motivation

Motivation theory suggests that motivation can be categorized into extrinsic and intrinsic motivation. According to Ryan and Deci (2001), intrinsic motivation refers to the driving force behind the behaviors that stems from the inherent satisfaction derived from the activity (e.g., internal enjoyment, need for self-growth), rather than external rewards (e.g., others’ admiration, material gains). Extrinsic motivations, such as fame, attractiveness, and wealth depend on the contingent reaction of others (Lee and Pounders, 2019). For instance, if a blogger shares activites out of a passion for social sharing (Reimer and Benkenstein, 2016) or self-expression to convey happiness (Meng et al., 2024; Zhang et al., 2024), their behaviors are driven by intrinsic motivation; conversely, if sharing is merely to gain financial rewards (Horng and Wu, 2020) or to attract admiration from others (Allard and White, 2015; Wang et al., 2024), it falls under extrinsic motivation. According to Derfler-Rozin and Pitesa (2020), we measure perceived intrinsic motivation by assessing consumers’ inferences about the reasons behind bloggers’ posting behaviors, and specifically whether the behaviors are driven by internal satisfaction such as taking challenges, learning, finding work enjoyable, etc.

On the one hand, hedonic behaviors are characterized by the pursuit of immediate gratification and sensory pleasure (Zhang et al., 2024; Elgammal et al., 2022). The pursuit of entertainment, and sensory stimulation provides limited enduring intrinsic value (Kasser and Ryan, 2001). Besides, when individuals engage in hedonic pursuits, they are often more susceptible to external temptation (Dhar and Wertenbroch, 2012), since the external rewards are typically easy means to provide immediate satisfaction (Kasser and Ryan, 2001). On the other hand, self-improvement activities focus on meaning and self-actualization, and they typically involve autonomous motives such as personal growth (Dong et al., 2021). According to Self-Determination Theory, actions driven by autonomous motives (e.g., personal growth) are perceived as intrinsically motivated (Derfler-Rozin and Pitesa, 2020). On the other hand, self-improvement behaviors require cognitive effort to continue finish the tasks while their rewards are often intrinsic (e.g., a sense of accomplishment) and delayed. Consumers tend to interpret persistence and sustained effort in self-improvement posts as evidence of genuine passion and intrinsic motivations (Dong et al., 2021).

The motivation perceptions influence the persuasiveness of social communications (Dickinger et al., 2008) and purchase intentions (Zhang and Ruan, 2024). Under conditions of information asymmetry, consumers often scrutinize posted content for potential ulterior motives (Santos et al., 2022; Wang et al., 2024). Prior research indicates that if a behavior is perceived as intrinsically motivated, it typically signals genuine, authentic and consistent in the eyes of consumers (Chu et al., 2023), thereby fostering more favorable evaluations toward the bloggers. In contrast, if a post is seen as extrinsically motivated, it often comes across as inauthentic and leads to psychological reactance (Zhang and Ruan, 2024). Lower perceived intrinsic motivation (e.g., audiences suspecting the blogger’s content is only for external gains) weakens the emotional connection and trust between the blogger and the audience, thereby reducing the positive evaluation of personal brand. Therefore, consumers may be more likely to identify with sharers posting self-improvement behaviors rather than hedonic ones, and thus we propose H2:

*H2: Perceived intrinsic motivation mediates the relationship between post type and personal brand evaluation.*

### 2.3 The moderating role of perceived social mobility

Social mobility refers to individuals’ subjective judgments and expectations regarding the likelihood of upward or downward shifts in their social class or socioeconomic status, reflecting perceived opportunities for advancement within the social hierarchy (Chen et al., 2024; Wang et al., 2022). Social mobility covers two dimensions, with one referring to individuals’ subjective evaluations of the possibility of achieving upward social mobility and the other to the structural opportunities within society that objectively facilitate such mobility. On the one hand, it refers to the individual’ s beliefs that they can improve their future socio-economic status through their own efforts (Chen et al., 2024). The higher likelihood of upward mobility in society implies that individuals are more likely to achieve a more promising future through their own efforts (Day and Fiske, 2019). On the other hand, it refers to the extent to which society allows members to rely on individual actions such as effort to achieve a better economic position (Wang et al., 2022).

High social mobility increases individuals’ opportunities to attain a higher quality of life through the improvement of their knowledge and skills (Chen et al., 2024). Research has shown that the extent of perceived social mobility shapes consumers’ attentiveness to self-improvement efforts. For instance, heightened perceptions of social mobility can drive parents to employ proactive strategies, such as preferring educational products that reinforce their children’s strengths, with the aim of attaining elevated social status (Chen et al., 2024). People increasingly recognize the value of self-improvement and admire those who strive through hard work. Diligence and ambition demonstrated by individuals are perceived as qualities of successful groups, aligning more closely with consumers’ expectations of ideal group members (Chen et al., 2024). Thus, when individuals share their self-improvement behaviors, which effectively signal the core traits of the ideal ingroup, consumers develop a stronger sense of identification with sharers. Secondly, a high-social-mobility environment enhances individuals’ endorsement of the belief that hard work leads to rewards. When social mobility is high, consumers are more likely to attribute self-improvement behaviors to genuine internal passion rather than external pressure (Lin et al., 2022). Therefore, in contexts of high social mobility, self-improvement behaviors are perceived as more intrinsically motivated, which in turn enhances consumers’ evaluations of the brand.

However, when consumers perceive lower social mobility, they lack confidence in the opportunity to achieve higher social status through own efforts (Chen et al., 2024). People with lower social mobility believe that the social class they live in has become a solid pattern, and no matter how hard they try, they cannot cross the social class they live in and be rewarded (Bjørnskov et al., 2013). The perceived association between self-improvement behaviors and the traits of an idealized ingroup is weakened. This is because consumers believe that such behaviors do not effectively help improve their current life circumstances. Consumers’ belief in the idea that hard work leads to higher social status diminishes, thereby weakening the perceived value of the sharer’ s self-improvement behaviors (Chen et al., 2024). Thus, when exposed to self-improvement behaviors, they no longer interpret such actions as driven by stronger intrinsic motivation. In contrast, they may interpret the sharer’ s self-improvement behaviors as responses to external pressures, aimed at securing better material benefits within their current social class (Siepmann et al., 2022). Thus, we propose that:

*H3: Perceived social mobility moderates the relationship between post type and personal brand evaluation. In high social mobility condition, sharing self-improvement (vs. hedonic) behaviors leads to higher personal brand evaluations; however, in low social mobility condition, such effect is attenuated.*

### 2.4 The moderating role of perceived social mobility

Perceived similarity is defined as an individual’s evaluation of the degree of psychological closeness between oneself and others with respect to values, behavioral patterns, or personal characteristics (Dang and Liu, 2024). Previous research has shown that people are more likely to identify themselves with those who are similar to them (Gelbrich et al., 2023). Despite the folk theory of opposites attracting, empirical evidence consistently shows that similarities foster attraction and in in-group relationships (Sunnafrank, 1983). People are attracted to people who are similar in many areas, including physical characteristics (Lauren Kim and Ellie Jin, 2023; Lou and Tse, 2021), personality traits, and shared experiences (Kim, 2024).

In high similarity condition, consumers may view the blogger as an in-group member. For example, demographically similar social relationships are perceived to be more reliable and motivated to help in group others (Lauren Kim and Ellie Jin, 2023; Lou and Tse, 2021). The ideality of self-improvement behaviors diminishes due to similarity, reducing the distinctiveness and appeal of such actions (Rodrigues et al., 2017). On the other hand, hedonic behaviors are more likely to resonate, as consumers have a positive view of themselves and in-group members (Heine et al., 2009). In other words, consumers will be more likely to approve and justify hedonic behaviors. In such conditions, the intrinsic motivation orientation behind self-improvement and hedonic behaviors are all in line with group identity, could be explained by the internal valuation for wellbeing and sharing daily lives, rather than for the sake of external validation. Therefore, consumers’ evaluations toward personal brands tend to be driven more by emotional resonance than by the pursuit of ideal traits. So, the distinction in the signal value of self-improvement and hedonic posts attenuates when perceived similarity with the blogger is high.

**Figure 1:**
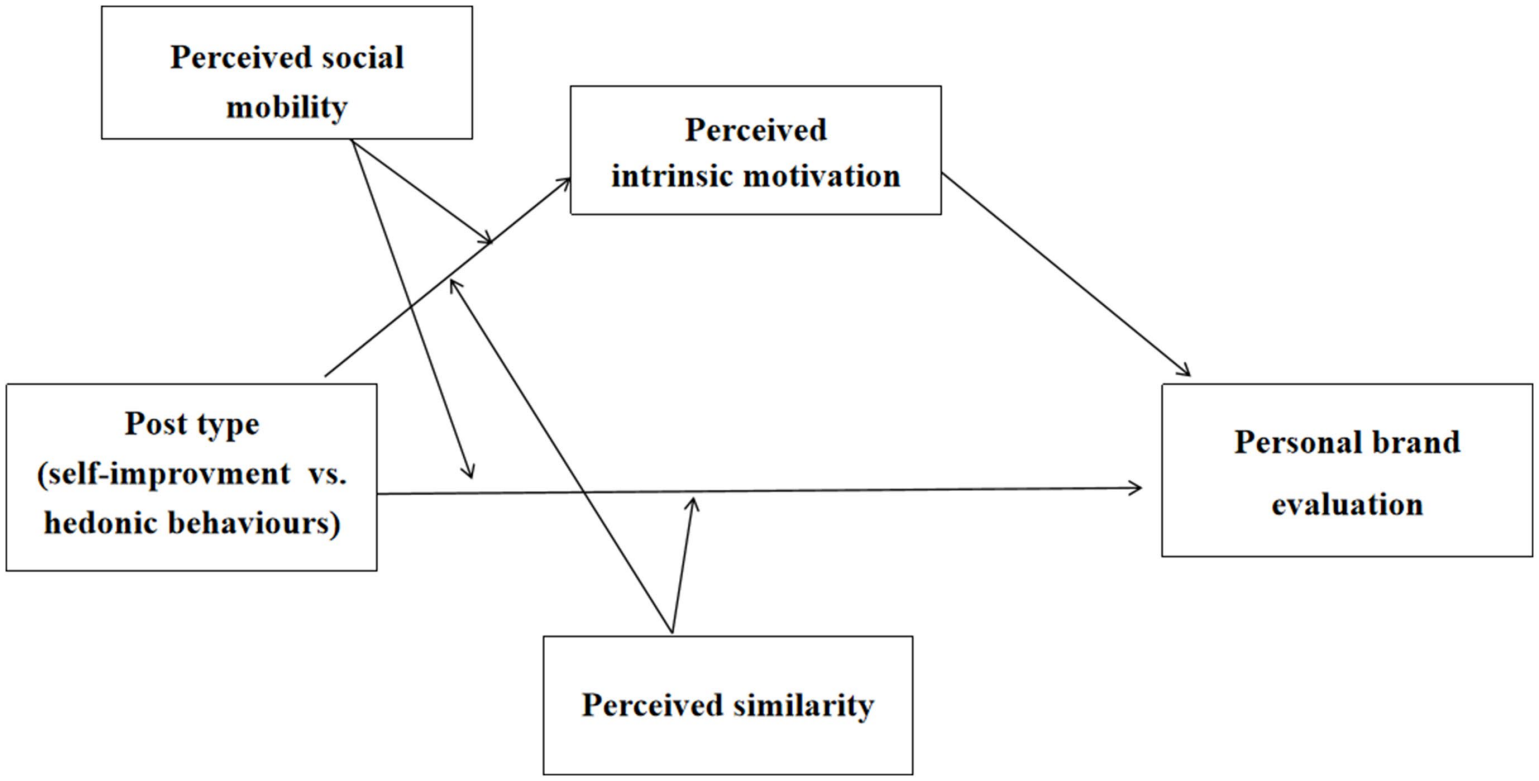
Theoretical framework.

However, in low similarity condition, their evaluations of the blogger tend to be based on value judgments rather than emotional resonance (Ahn et al., 2021). The bloggers may seem as outsiders with distinct traits and lifestyles that contrast with users’ own reality (Shehzala et al., 2024; Venciute et al., 2023). If the bloggers share self-improvement behaviors such as skill development, it sends out a positive signal as a progressive attribute to achieve a better state (Wang et al., 2024). Even if consumers are not the bloggers’ fans, they would value self-improvement behaviors as a socially desirable sign worth emulating, and form positive evaluations (Zhang et al., 2024). By contrast, if the blogger shares hedonic behaviors (e.g., recreational activities), users are more likely to interpret it as mere seeking hedonic wellbeing without the pursuit of intrinsic life goals (Ryan and Deci, 2001). The focus on immediate gratification may also fail to provide similar internal value that consumers trust, deviating the socially expectations of positive image of their ideal group, and thus lowering personal brand evaluations. Thus, we propose that:

*H4: Perceived similarity moderates the relationship between post type and personal brand evaluation. Specifically, in low similarity condition, sharing self-improvement (vs. hedonic) behaviors leads to higher personal brand evaluation; however, in high similarity condition, such effect is attenuated (Figure 1).*

## 3 Methodology

### 3.1 Study 1: the effect of post type on perceived intrinsic motivation

This study employs a 3 (post type: hedonic, mixed, self-improvement behaviors) factorial between-subjects design. The purpose of Study 1 is to explore the effect of post type (hedonic vs. self-improvement behaviors) on positive personal brand evaluation such as perceived intrinsic motivation.

#### 3.1.1 Pilot test

To ensure our stimuli aligned with actual social media behaviors, we conducted a pre-test to develop experimental materials. In the first step, we asked two marketing managers, two academic experts, and ten university students to list five checklists of self-improvement and hedonic behaviors they usually see on social media posts. In the hedonic behavior checklist, activities such as watching movies (85.7%, 12 out of 14), walking (78.6%, 11 out of 14), sleeping (78.6%, 11 out of 14), and playing games (71.4%, 10 out of 14) were mentioned most frequently. In the self-improvement behavior checklist, activities such as exercising (92.9%, 13 out of 14), reading (85.7%, 12 out of 14), learning software skills (71.4%, 10 out of 14) and cooking tutorial learning (64.3%, 9 out of 14) were reported more frequently. In the second step, we then selected the most cited behaviors and designed the related posts. We asked 60 participants to give ratings for each post such as: “Does this post look like something you’d actually see on social media?,” “To what extent does this post resemble real media content?,” and “How typical is the post of what’s usually shared on social media?” (*α* = 0.89). We selected posts with average ratings over 5 to ensure our stimuli are ground. Finally, we constructed the experimental materials. All stimuli were strictly controlled to have identical length, including the number of English characters, punctuation marks, and linguistic expressions within it. And for sharing frequency, all stimuli included a fictional share count, and consumers only see 4 recent posts as representations. Besides, in terms of tone, by using AI to assess the positivity of the language between the two groups, we found that after conducting consecutive assessments five times, there were no significant differences in the overall convey upbeat including positivity, enthusiasm and a cheerful tone (*ps* > 0.05).

#### 3.1.2 Participants

Participants were recruited through the MTurk. Each participant provided their informed consent online and received a small monetary compensation. A total of 250 questionnaires were distributed. After removing four that failed the attention check, 246 valid sample was retained. Among them, 51.2% of participants were female, with a mean age of 42.34 (SD_age_ = 10.29).

#### 3.1.3 Methods and procedures

A fictional blogger nicknamed “Luna Parker” was created for this study. Firstly, participants were randomly assigned to one of the three groups to receive the manipulation for post type. In the hedonic condition, participants read four recent posts related to hedonic behaviors such as: “Movie night + popcorn = my happy place,” “Slept till 10 a.m.… No alarms, just peace,” “Walked to park for ice cream—sunshine tastes good” and “Played board games with friends all evening~Super fun.” In the self-improvement condition, the activities include: “Morning yoga flow, small steps for better me,” “Night reading for project. Growth mode on,” “Learned new Excel trick today — Small wins add up” and “Followed a cooking tutorial today and unlocked a new skill ~Super fun.” Under the mixed condition, the influencer shared posts that included both hedonic and self-improvement behaviors, including: “Movie night + popcorn = my happy place,” “Night reading for project. Growth mode on,” “Walked to park for ice cream—sunshine tastes good” and “Followed a cooking tutorial today and unlocked a new skill ~Super fun”(see Appendix A).

After reading the recent posts, we measured perceived intrinsic motivation through the following items: “The blogger is interested in the job because of the satisfaction he or she would experience from taking on interesting challenges,” “The blogger is interested in the job because of the satisfaction he or she would experience from being successful in a challenging and fun task,” “The blogger is interested in the job because he or she derives much pleasure from learning new things” and “The blogger is interested in the job because he or she finds the work itself enjoyable” (*α* = 0.91; adapted from Derfler-Rozin and Pitesa, 2020).

Then, participants were asked to complete the manipulation check of post type, by rating their perceptions of the posts, such as “To what extent do you think the blogger’ s posts reflect his self-improvement behaviors,” and “To what extent do you think the blogger’ s posts reflect his hedonic behaviors.” In addition, we assessed participants’ perceptions of post realism by asking them: “To what extent do you think the posts shared by the bloggers were realistic” (1 = not at all, 7 = very much; adapted from Huang et al., 2024). Finally, participants provided their demographic information including age, gender and education.

#### 3.1.4 Results

##### 3.1.4.1 Manipulation check

One-way ANOVA indicates that participants in the self-improvement condition report significantly greater perceptions of self-improvement attributes in the posts compared with those in the other two conditions (M_self-improvement_ = 5.90, SD = 1.14; M_mixed_ = 5.41, SD = 1.10; M_hedonic_ = 4.24, SD = 1.75; *F*(2, 242) = 31.71, *p* < 0.001). The participants in the hedonic condition perceive higher hedonic attributes in the posts than the other two conditions (M_hedonic_ = 5.73, SD = 1.12; M_mixed_ = 5.30, SD = 1.15; M_self-improvement_ = 4.37, SD = 1.71; *F*(2, 243) = 21.76, *p* < 0.001). Meanwhile, no significant group differences were found in perceived realism of the content across the three conditions (M_hedonic_ = 5.48, SD = 1.25; M_mixed_ = 5.45, SD = 1.14; M_self-improvement_ = 5.36, SD = 1.34; *F*(2, 243) = 0.22, *p* = 0.803). This indicates that the experimental materials closely resemble real-life contexts. Thus, the manipulation of post type is successful.

##### 3.1.4.2 Perceived intrinsic motivation

The one-way ANOVA shows a significant difference among the three groups in perceived intrinsic motivation (M_hedonic_ = 4.78, SD = 1.50; M_mixed_ = 5.19, SD = 1.17; M_self-improvement_ = 5.44, SD = 1.00; *F*(2, 243) = 5.69, *p* = 0.004).

#### 3.1.5 Discussion

Study 1 collected data from U. S. sample. The results demonstrate that different types of post content lead to significant differences in consumers’ positive evaluations for intrinsic motivation of the personal brands. We then moved on the examine the effects of post type on personal brand evaluation using some behavioral intention index in Study 2.

### 3.2 Study 2: the main effect and the mediating effect

This study employs a 3 (post type: hedonic, mixed, self-improvement behaviors) factorial between-subjects design. The purpose of Study 2 is to explore the effect of post type (hedonic vs. self-improvement behaviors) on personal brand evaluation (H1) and the mediating effect of perceived intrinsic motivation (H2). Besides, we also want to see how Chinese consumers respond to posts with mixed hedonic and self-improvement activities. In addition, we aim to rule out confounding variables including social norm, perceived extrinsic motivation, realism, general preference for hedonic and self-improvement behaviors, and perceived authenticity.

#### 3.2.1 Participants

Participants were recruited through the Credamo (www.credamo.com, an online panel based in China). Each participant provided their informed consents online and was paid for a small monetary compensation. A total of 250 questionnaires were distributed. After removing ten that failed the attention check, 240 valid sample was retained. Among them, 62.1% of the participants were female, 66.7% had a bachelor’ s degree and 82.6% of the participants were between the ages of 21 and 40.

#### 3.2.2 Methods and procedures

A fictional blogger nicknamed “Luna Parker” was created for this study. Firstly, participants were randomly assigned to one of the three groups to receive the manipulation for post type. The manipulation materials were similar to Study 1 (see Appendix A). After that, participants were asked to report their personal brand evaluation, perceived intrinsic motivation, perceived extrinsic motivation. Specifically, personal brand evaluation was measured through three items: “How likely would you give a like to this blogger’s content,” “How likely would you follow this blogger and see more contents” and “How likely would you purchase products or engage in activities promoted by this blogger” (*α* = 0.83; adapted from Naylor et al., 2012). Perceived intrinsic motivation was measured similar to Study 1 (*α* = 0.79; adapted from Derfler-Rozin and Pitesa, 2020). In addition, we then measured some confounding variables. Perceived extrinsic motivation was measured through the following items: “To what extent do you think the blogger wants to make a financial return,” “To what extent do you think the blogger aims to gain admiration from others,” “To what extent do you think the blogger wants to gain prestige and reputation” (*α* = 0.79; adapted from Pelletier et al., 1997). In addtion, perceived authenticity was measured by asking participants to what extent they perceived the blogger as real and authentic (Jin et al., 2022). Social norms perception was measured by asking to what extent they agreed that the blogger’s posts conform to social norms/ meet social expectations (*α* = 0.72; adapted from Kim and Seock, 2019).

Then, participants were asked to complete the manipulation check of post type. In addition, we assessed participants’ perceptions of post realism by asking them: “To what extent do you think the posts shared by the bloggers were realistic” (1 = not at all, 7 = very much; adapted from Huang et al., 2024). Finally, participants provided their demographic information including age, gender, education, and their general preference for hedonic and self-improvement behaviors.

#### 3.2.3 Results

*Manipulation check.* One-way ANOVA indicates that participants in the self-improvement condition report significantly greater perceptions of self-improvement attributes in the posts compared with those in the other two conditions (M_self-improvement_ = 6.16, SD = 0.82; M_mixed_ = 4.68, SD = 1.43; M_hedonic_ = 2.78, SD = 1.41; *F*(2, 237) = 146.786, *p* < 0.001). The participants in the hedonic condition perceive higher hedonic attributes in the posts than the other two conditions [M_hedonic_ = 6.08, SD = 1.07; M_mixed_ = 4.88, SD = 1.43; M_self-improvement_ = 3.44, SD = 1.57; *F*(2, 237) = 74.13, *p* < 0.001]. Meanwhile, no significant group differences were found in perceived realism of the content across the three conditions [M_hedonic_ = 5.68, SD = 0.91; M_mixed_ = 5.96, SD = 0.99; M_self-improvement_ = 5.76, SD = 0.75; *F*(2, 237) = 2.20, *p* = 0.110]. This indicates that the experimental materials closely resemble real-life contexts. Thus, the manipulation of post type is successful.

##### 3.2.3.1 Main effect

**Figure 2:**
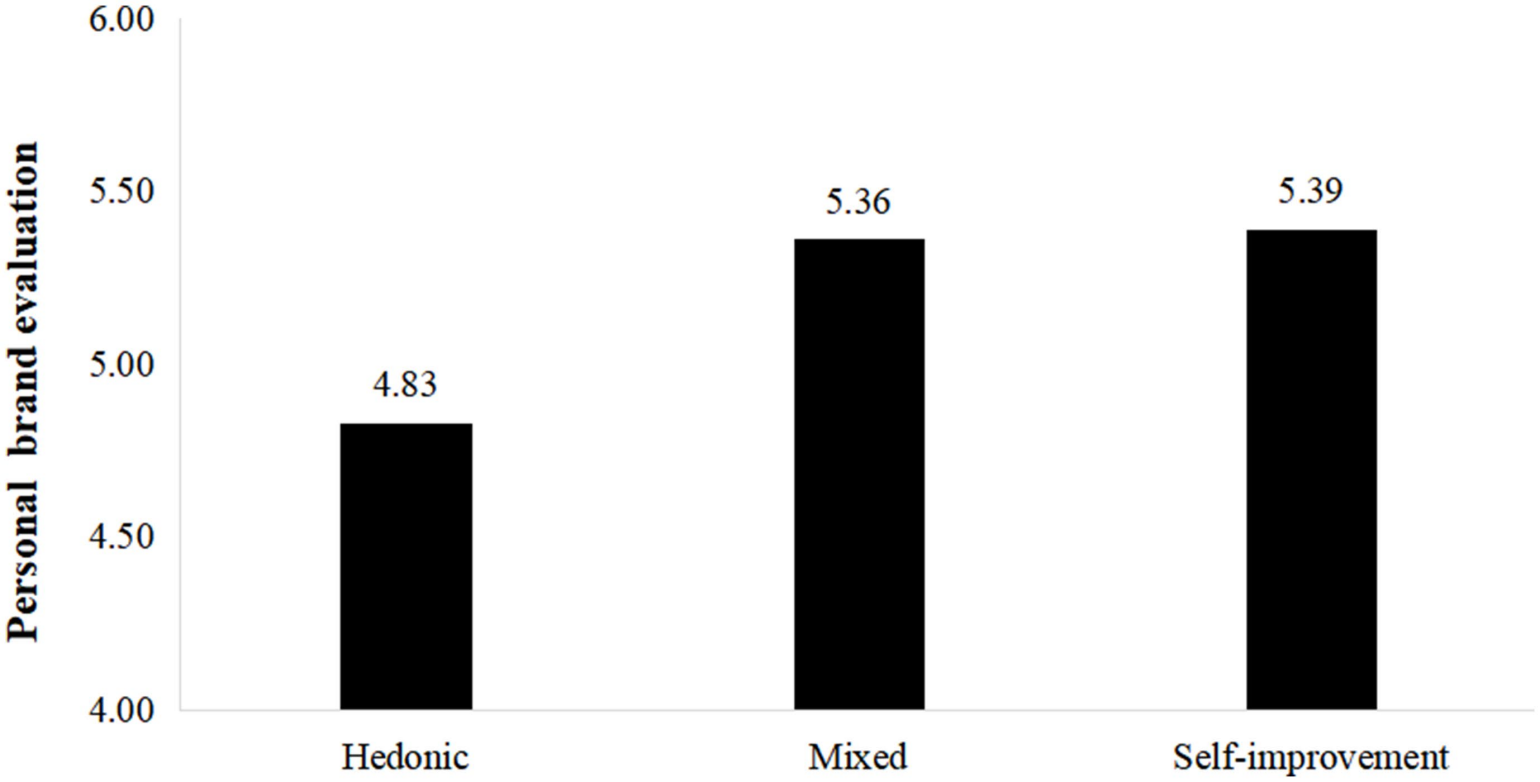
One-way ANOVA analysis for study 2.

The one-way ANOVA shows a significant difference in personal brand evaluations among the three groups. Specifically, the self-improvement group reports significantly higher evaluations than either of the other two groups [M_hedonic_ = 4.83, SD = 1.40; M_mixed_ = 5.36, SD = 0.92; M_self-improvement_ = 5.39, SD = 0.93; *F*(2, 237) = 6.52, *p* = 0.002; see Figure 2]. Specifically, self-improvement group has significantly higher personal brand evaluation than the hedonic group [M_self-improvement_ = 5.39, SD = 0.93; M_hedonic_ = 4.83, SD = 1.40; t (158) = 3.00, *p* = 0.003]. Similarly, there was a significant difference between the hedonic group and the mixed group in their evaluations of the brand [M_hedonic_ = 4.83, SD = 1.40; M_mixed_ = 5.36, SD = 0.92; t (158) = −2.83, *p* = 0.005]. However, there was no significant difference in personal brand evaluations between the self-improvement group and the mixed group [M_self-improvement_ = 5.39, SD = 0.93; M_mixed_ = 5.36, SD = 0.92; t (158) = 0.23, *p* = 0.82]. Thus, H1 is supported.

##### 3.2.3.2 Mediating effect

To validate the mediation role of perceived intrinsic motivation, we conducted a mediation analysis using bootstrapping (Model 4, based on 5,000 samples; Hayes, 2013). Results demonstrate that perceived intrinsic motivation mediates the effect of post type on personal brand evaluation (Indirect effect = 0.25, s.e. = 0.06, 95% CI = [0.1320, 0.3809]). Thus, H2 is supported. Additionally, after controlling for social norm, perceived extrinsic motivation, general preference for hedonic and self-improvement behaviors, and perceived authenticity, the mediating effect of perceived intrinsic motivation remains unchanged (Indirect effect = 0.08, s.e. = 0.04, 95% CI = [0.0066, 0.1611]).

#### 3.2.4 Discussion

Study 2 reveals that consumers demonstrate better evaluations for bloggers sharing self-improvement behaviors rather than hedonic behaviors, supporting H1. Besides, the underlying mechanism of perceived intrinsic motivation is verified and provides support for H2. Additionally, Study 2 rules out the potential confounding effects of social norm, perceived extrinsic motivation, realism, general preference for hedonic and self-improvement behaviors, and perceived authenticity. The results of this study indicate that there is a significant difference in personal brand evaluations between the hedonic group and the self-improvement group, Thus, H1 is supported. Moreover, we find a nonsignificant difference between the mixed group and the self-improvement group. This may be because the inclusion of hedonic content increase authenticity that offsets the potential dilution of the intrinsic motivation induced by self-improvement posts, and ultimately allowing the mix group to match the pure self-improvement group’s evaluation.

### 3.3 Study 3: the moderating effect of perceived social mobility

This study employs a 2 (perceived social mobility: high vs. low) * 2 (post type: hedonic vs. self-improvement behaviors) between-subjects design. We intend to rule out the confounding effects of positive emotion and perceived usefulness. Moreover, this study aims to explore the moderating effect of perceived social mobility (H3).

#### 3.3.1 Participants

Participants were recruited from Credamo.^1^ Each participant provided their informed consent online and was paid a small monetary compensation for completing the questionnaire. A total of 300 questionnaires were distributed. After removing those that failed the attention check, 291 valid questionnaires were retained. In this experiment, 67% of participants were female, 81.8% of the participants were between the ages of 21 and 40, and 75.6% of the participants had a bachelor’ s degree.

#### 3.3.2 Methods and procedures

Participants were randomly assigned to one of four scenarios. Firstly, we manipulated perceived social mobility by providing them with an excerpt from a recent report on China’ s social development, but the content varied across conditions. In the high social mobility condition, the report titles “Moving Toward a Higher Place.” The report emphasizes that in the current social environment, 66% of individuals can achieve higher incomes than their parents through hard work. In the low social mobility condition, the report titles “Moving to a Higher Place?.” The report emphasizes that in the current social environment, many individuals from lower social strata are unable to move up the social ladder and are likely to remain in the same social class as their parents. Then, participants were presented with one blog posted by a social media influencer called Tang. In the hedonic condition, Tang recommends four hedonic videos he recently watched, followed by his description like: “Lately, I’ve been fully immersed in entertainment. I’ve watched all sorts of hilarious videos to just enjoy the moment and forget all my troubles.” However, in the self-improvement condition, Tang posts four videos related to self-improvement, followed by a description like: “Lately, I’ve been deeply engaged in studying, watching various educational videos to enhance my skills and knowledge” (see Appendix B).

After reading the recent posts, participants reported their personal brand evaluation and perceived intrinsic motivation. Personal brand evaluation was measured through three items: “What is your overall impression of the blogger,” “To what extent do you like this blogger,” and “How do you feel about this blogger” (*α* = 0.91; adapted from Valsesia and Diehl, 2022). Perceived intrinsic motivation was measured similarly to Study 1 (*α* = 0.71; adapted from Derfler-Rozin and Pitesa, 2020). Next, we measured participants’ positive emotion and perceived usefulness of the information. Participants’ positive emotion was measured through the following items: “I am currently feeling depressed (reverse-coded),” “I am currently feeling happy,” “I am currently in a bad mood (reverse-coded),” “I am currently feeling unhappy (reverse-coded)” and “I am currently feeling joyful” (*α* = 0.89; adapted from Han et al., 2022). Perceived usefulness of the information was measured through the following items: “The content shared by blogger Tang is helpful to me,” “The content shared by blogger Tang brings me many benefits” and “The content shared by blogger Tang helps to improve my abilities” (*α* = 0.90; adapted from Siagian et al., 2022).

Finally, participants completed the manipulation checks for post type and perceived social mobility. The manipulation check for post type was assessed using the following items: “To what extent do you think this post shared by blogger Tang reflects his self-improvement behaviors (e.g., learning, personal growth)” and “To what extent do you think this post shared by blogger Tang reflects his hedonic behaviors (e.g., entertainment, sensory pleasure).” Participants were then asked to rate their perceived social mobility through the following items: “I have a lot of opportunities for advancement in society,” “I can change my social class,” “I can be richer if I want to be,” “I′ m unlikely to improve my social status soon (reverse coded),” “I may be stuck in my current social class (reverse coded),” “I don’ t have many opportunities to improve my position in society (reverse coded)“and “I have many choices in life” (*α* = 0.95; adapted from Day and Fiske, 2016). Lastly, participants provided their demographic information such as age, gender and education.

#### 3.3.3 Results

##### 3.3.3.1 Manipulation checks

An ANOVA on perceived self-improvement attribute of the content yielded a significant main effect of post type [*F*(1, 287) = 1982.31, *p* < 0.001, partial *η*^2^ = 0.874, Cohen’ s f^2^ = 6.874]. Specifically, participants in the self-improvement content condition perceived the content as having stronger self-improvement attributes (M = 6.36, SD = 0.75) than those in the hedonic content condition (M = 1.93, SD = 0.93). An ANOVA on perceived hedonic attribute of the content yielded a significant main effect of post type [*F*(1, 287) = 1551.09, *p* < 0.001, partial *η*^2^ = 0.844, Cohen’ s f^2^ = 5.410. Specifically, participants in the hedonic content condition perceived the content as having stronger hedonic attributes (M = 6.31, SD = 0.73) than those in the self-improvement content condition (M = 1.93, SD = 1.12). Meanwhile, no significant differences were found between the two groups in their perception of the positive emotions conveyed by the post content [M_self-improvement_ = 5.62, SD = 0.96; M_hedonic_ = 5.50, SD = 1.19; t (289) = 0.99, *p =* 0.323]. Similarly, another ANOVA on the perceived social mobility revealed a significant main effect of social mobility [*F*(1, 287) = 300.90, *p* < 0.001, partial *η*^2^ = 0.512, Cohen’s f^2^ = 1.049]. Specifically, participants in the high social mobility condition reported stronger perceptions of social mobility (M = 5.07, SD = 1.08) than did those in the low social mobility condition (M = 2.85, SD = 1.10). Thus, the manipulations of perceived social mobility and post type are successful.

##### 3.3.3.2 Main effect

An independent t-test reveals that the self-improvement group has significantly higher personal brand evaluation than the hedonic group (M_self-improvement_ = 5.70, SD = 0.89; M_hedonic_ = 4.85, SD = 1.23; t (289) = 6.79, *p* < 0.001). Thus, H1 is supported.

##### 3.3.3.3 Mediating effect

To validate the mediation role of perceived intrinsic motivation orientation, we conducted a mediation analysis using bootstrapping (Model 4, based on 5,000 samples; Hayes, 2013). We set post type as the independent variable, personal brand evaluation as the dependent variable, and perceived intrinsic motivation as the mediating variable. Results demonstrate that perceived intrinsic motivation mediates the effect of post type on personal brand evaluation (Indirect effect = 1.30, s.e. = 0.13, 95% CI = [1.0541, 1.5481]). After controlling for perceived usefulness, the mediating effect remains unchanged (Indirect effect = 0.37, s.e. = 0.08, 95% CI = [0.2137, 0.5513]). Thus, H2 is supported.

##### 3.3.3.4 Moderating effect of perceived social mobility

**Figure 3:**
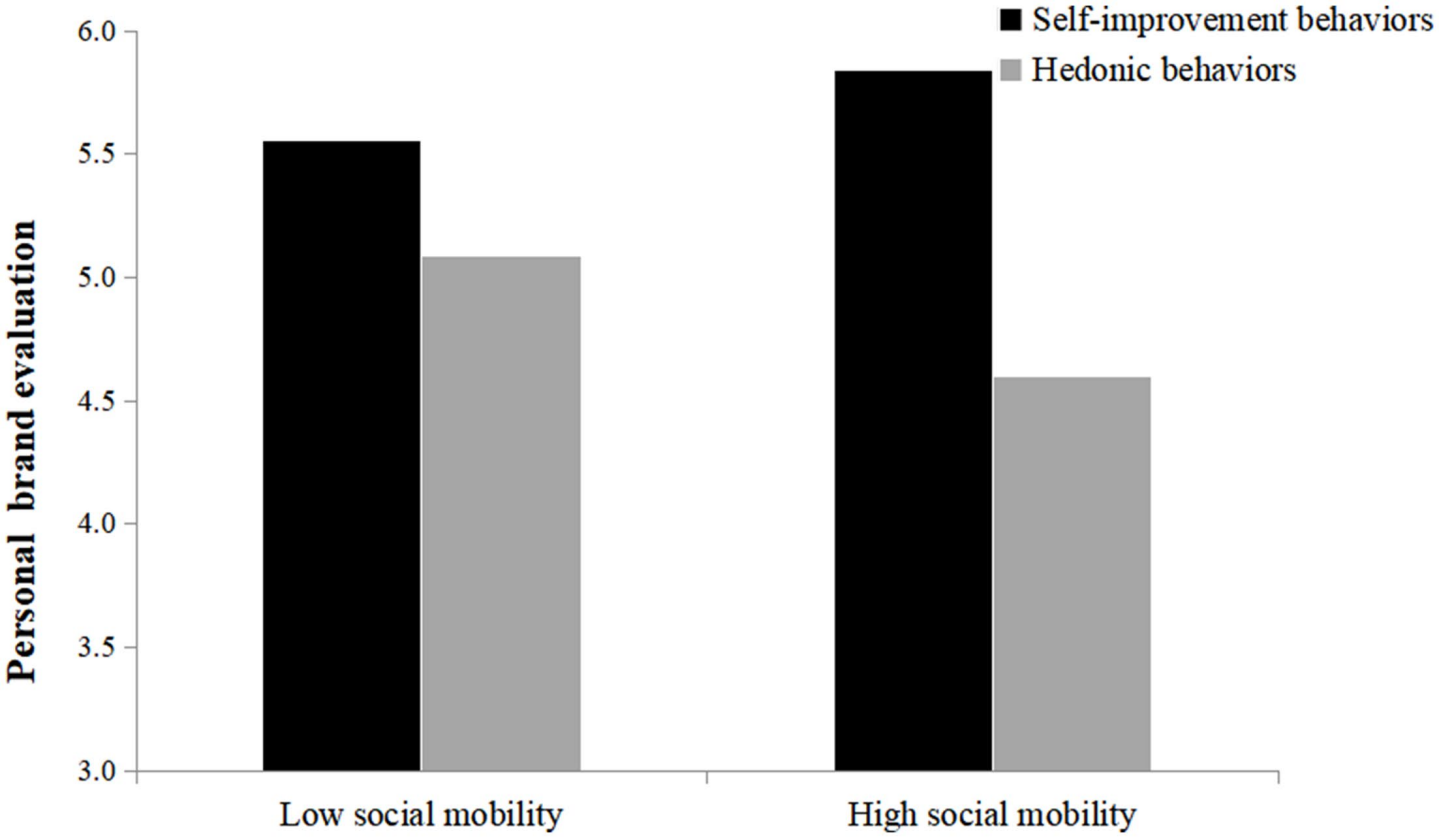
The moderating effect of perceived social mobility.

An ANOVA on personal brand evaluation yielded a significant interaction effect between social mobility and post type [*F*(1, 287) = 9.35, *p* = 0.002, partial *η*^2^ = 0.032]. The follow-up simple effects test showed that a significant difference between different post types on personal brand evaluation for participants in the high social mobility condition [M_self-improvement_ = 5.84, SD _self-improvement_ = 0.70; M_hedonic_ = 4.60, SD_hedonic_ = 1.34; *F*(1, 287) = 55.48, *p* < 0.001, partial *η*^2^ = 0.147]. However, at low level of social mobility, the effect of post type on personal brand evaluation is reduced [M_self-improvement_ = 5.56, SD_self-improvement_ = 1.04; M_hedonic_ = 5.08, SD_hedonic_ = 1.07; *F*(1, 287) = 7.253, *p* = 0.07, partial *η*^2^ = 0.025; Figure 3]. Thus, H3 is supported.

##### 3.3.3.5 Moderated mediation analysis

A 2 × 2 ANOVA on perceived intrinsic motivation showed that the interaction effect [*F*(1, 287) = 4.65, *p* = 0.030, partial *η*^2^ = 0.016]. We also conducted a moderated mediation analysis using the PROCESS Model 8 (Hayes, 2013), with a bootstrap sample of 5,000, in which post type (0 = hedonic behaviors; 1 = self-improvement behaviors) as the independent variable, perceived social mobility (0 = low; 1 = high) as the moderator, perceived intrinsic motivation was the mediator, and personal brand evaluation was the dependent variable. The results confirm a significant moderated mediation effect (Effect = 0.34, s.e. = 0.16, 95% CI: [0.0440, 0.6673]). In particular, there is a significant mediation effect of perceived intrinsic motivation in high social mobility condition (Effect = 1.44, s.e. = 0.16, 95% CI: [1.1414, 1.7610). But in the low social mobility condition, the effect is attenuated (Effect = 1.10, s.e. = 0.14, 95% CI: [0.8408, 1.3653]).

#### 3.3.4 Discussion

Study 3 verifies the moderating effect of perceived social mobility, supporting H3. Specifically, in contexts of high social mobility, self-improvement content shared by personal brands is perceived as reflecting stronger intrinsic motivation. Such perceptions resonate with the value orientation emphasized in high-mobility societies, thereby enhancing consumers’ evaluations of the brand. Conversely, when social mobility is perceived to be low, consumers do not differentiate between self-improvement and hedonic content in terms of intrinsic motivation, resulting in no significant differences in brand evaluations. In addition, we rule out the confounding effects including positive emotion and perceived usefulness.

### 3.4 Study 4: the moderating effect of perceived similarity

This study employs a 2 (perceived similarity: high vs. low) * 2 (post type: hedonic vs. self-improvement behaviors) factorial between-subjects design. The purpose of Study 4 is to replicate the findings of the prior studies and explore the moderating effect of perceived similarity (H4). In addition, we aim to rule out the potential confounding effect of perceived extrinsic motivation, realism, perceived authenticity, general preference for hedonic and self-improvement behaviors, and perceived effort.

#### 3.4.1 Participants

Participants were recruited from Credamo (www.credamo.com). Each participant provided their informed consent online and was paid a small monetary compensation for completing the task. A total of 300 questionnaires were distributed. After removing 2 that failed the attention check, 298 valid questionnaires were retained. Among them, 60.1% of participants were female, with a mean age of 34.02 (SD_age_ = 7.40), and 73.2% of the participants had a bachelor’ s degree.

#### 3.4.2 Methods and procedures

First, participants reported their personal information, including gender, age, place of residence, and education. Then, participants were shown the profile of a social media influencer nicknamed “Baaba.” To manipulate similarity, we drew on Naylor and Lamberton’ s method (Naylor et al., 2011). Perceived similarity was manipulated by varying the extent to which the influencer’s personal information matched that of the participants. In the high similarity condition, the influencer’s age, gender, and place of residence were completely identical to those of the participants. However, in the low similarity condition, the influencer is 42 years old, living in Shanxi and having the opposite gender with the participants.

Next, participants were then randomly assigned either hedonic or self-improvement scenarios, where they read posts from the influencer. The manipulation materials were similar to Study 1 (see Appendix A). After that, participants were asked to report their personal brand evaluation, perceived intrinsic motivation, perceived extrinsic motivation. Similar to Study 2, personal brand evaluation (*α* = 0.90; adapted from Naylor et al., 2012), perceived intrinsic motivation (*α* = 0.86; adapted from Derfler-Rozin and Pitesa, 2020), perceived authenticity (Jin et al., 2022), general preference for hedonic and self-improvement behaviors, and perceived extrinsic motivation (*α* = 0.80; adapted from Pelletier et al., 1997) were measured. Subsequently, perceived effort was measured by asking participants to evaluate the level of effort invested by the influencer (adapted from Huang et al., 2024). In addition, we assessed participants’ perceptions of post realism similar to Study 1 (Huang et al., 2024). Finally, participants completed the manipulation checks for post type and perceived similarity. The manipulation check for post type was similar to prior studies. Perceived similarity was measured by asking participants: “I think the blogger is not like me at all (reverse coded),” “I believe the influencer and I share many commonalities,” “I perceive the influencer and myself as completely dissimilar (reverse coded)” and “I think the blogger is similar to me in many ways” (*α* = 0.89; Naylor et al., 2011).

#### 3.4.3 Results

##### 3.4.3.1 Manipulation checks

An ANOVA on perceived self-improvement attribute of the content yields a significant main effect of post type [*F*(1, 294) = 1062.55, *p* < 0.001, partial *η*^2^ = 0.783, Cohen’ s f^2^ = 3.61]. Specifically, participants in the self-improvement condition perceive the content as having stronger self-improvement attributes (M = 6.07, SD = 0.91) than those in the hedonic condition (M = 2.28, SD = 1.15). Similarly, perceived hedonic attribute of the content yields a significant main effect of post type [*F*(1, 294) = 571.10, *p* < 0.001, partial *η*^2^ = 0.660, Cohen’ s f^2^ = 1.94], and participants in the hedonic condition perceive the content as having stronger hedonic attributes (M = 6.19, SD = 0.82) than those in the self-improvement condition (M = 2.89, SD = 1.47). Besides, perceived similarity revealed a significant main effect of similarity [*F*(1, 294) = 207.06, *p* < 0.001, partial *η*^2^ = 0.413, Cohen’s f^2^ = 0.70]. Participants in the high similarity condition report stronger perceptions of similarity (M = 5.15, SD = 1.00) than those in the low similarity condition (M = 3.19, SD = 1.35). Meanwhile, no significant differences in perceived realism of the content were found between the two conditions [M_hedonic_ = 5.84, SD = 0.95; M_self-improvement_ = 5.97, SD = 0.86; t (296) = −1.29, *p* = 0.197]. Thus, the manipulations of similarity and post type are successful.

##### 3.4.3.2 Main effect

An independent t-test reveals that the self-improvement group has significantly higher personal brand evaluation than those in hedonic group [M_self-improvement_ = 5.37, SD = 1.03; M_hedonic_ = 4.23, SD = 1.54; t (296) = 7.55, *p* < 0.001]. Thus, H1 is supported.

##### 3.4.3.3 Mediating effect

To validate the mediation role of perceived intrinsic motivation, we conducted a mediation analysis using bootstrapping (Model 4, based on 5,000 samples). We set post type as the independent variable, personal brand evaluation as the dependent variable, and perceived intrinsic motivation as the mediating variable. Results demonstrate that perceived intrinsic motivation mediates the effect of post type on personal brand evaluation (Indirect effect = 0.64, s.e. = 0.10, 95% CI = [0.4544, 0.8537]). Thus, H2 is supported. Additionally, after controlling for perceived extrinsic motivation, perceived authenticity, general preference for hedonic and self-improvement behaviors and perceived effort, the results remain unchanged (Indirect effect = 0.07, s.e. = 0.04, 95% CI = [0.0036, 0.1686]).

##### 3.4.3.4 Moderating effect of perceived similarity

**Figure 4:**
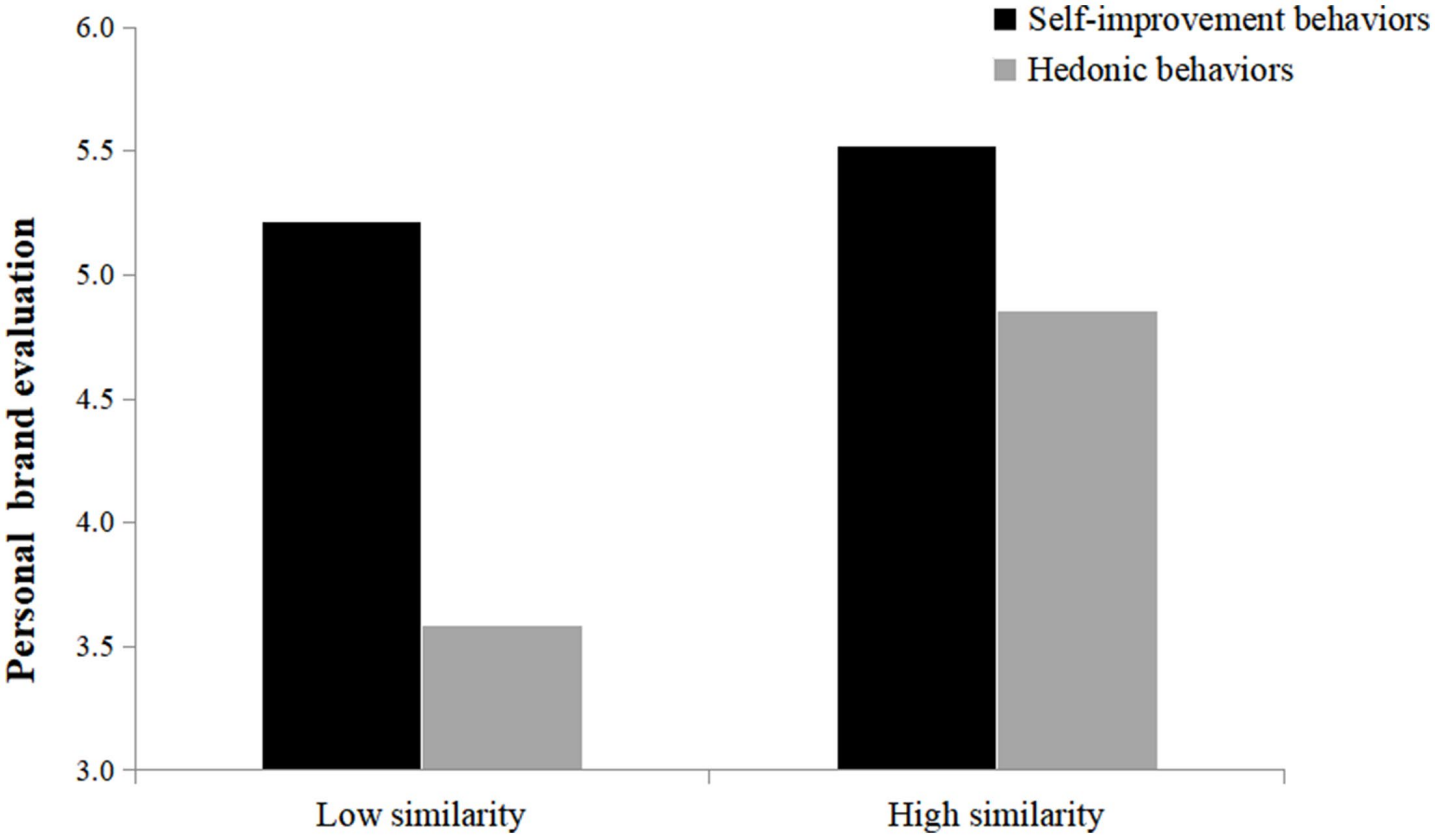
The moderating effect of perceived similarity.

An ANOVA on personal brand evaluation yields a significant interaction effect between perceived similarity and post type [*F*(1, 294) = 11.21, *p* < 0.001, partial *η*^2^ = 0.037]. The follow-up simple effects test shows a significant effect for participants in the low similarity condition [M_self-improvement_ = 5.21, SD _self-improvement_ = 1.03; M_hedonic_ = 3.60, SD_hedonic_ = 1.52; *F*(1, 294) = 64.82, *p* < 0.001, partial *η*^2^ = 0.181]. However, for participants in the high similarity condition, the positive effect of self-improvement is attenuated [M_self-improvement_ = 5.52, SD_self-improvement_ = 1.02; M_hedonic_ = 4.86; SD_hedonic_ = 1.29; *F*(1, 294) = 11.00, *p* < 0.001, partial *η*^2^ = 0.036; see Figure 4]. Thus, H4 is supported.

##### 3.4.3.5 Moderated mediation analysis

An ANOVA on perceived intrinsic motivation yielded a significant interaction effect between perceived similarity and post type [*F*(1, 294) = 4.65, *p* = 0.032, partial *η*^2^ = 0.016]. We also conducted a moderated mediation analysis using the PROCESS Model 8 (Hayes, 2013), with a bootstrap sample of 5,000, in which post type (0 = hedonic; 1 = self-improvement) as the independent variable, perceived similarity (0 = low; 1 = high) as the moderator, perceived intrinsic motivation was the mediator, and personal brand evaluation as the dependent variable. The results confirm a significant moderated mediation effect (Effect = − 0.28, s.e. = 0.14, 95% CI = [−0.5729, − 0.0252]). In particular, the mediating effect is significant in low similarity condition (Effect = 0.72, s.e. = 0.14, 95% CI = [0.4639, 0.9913]), while the mediating effect is attenuated in the high similarity condition (Effect = 0.43, s.e. = 0.10, 95% CI = [0.2395, 0.6449]). Therefore, H4 is supported.

#### 3.4.4 Discussion

Study 4 reveals a significant moderating effect of perceived similarity between audience and the blogger. Specifically, the positive effect of self-improvement (vs. hedonic) on personal brand evaluation significantly attenuates when the audience shares higher intrapersonal similarity to the blogger. This may be because in the high similarity condition, consumers’ tendency to identify and make associations with bloggers is high, regardless of the actual behaviors they share. In addition, we also rule out the confounding effect of perceived extrinsic motivation, perceived effort, realism, perceived authenticity, and general preference for hedonic and self-improvement behaviors.

## 4 General discussions

### 4.1 Theoretical contributions

Our research makes several important contributions to the social media influencers literature. Firstly, this article contributes to the impression formation research by introducing a novel classification of shared contents (i.e., hedonic and self-improvement behaviors). As two types of universal paths toward wellbeing, hedonic behaviors pursue immediate sensory pleasure and emotional wellbeing; whereas self-improvement behaviors focus on personal development for eudaimonic wellbeing. While prior research has primarily focused on external stimuli that promote self-improvement vs. hedonic choices (Allard and White, 2015; Grewal et al., 2022; Zhang et al., 2024), or how these two types of behaviors influence sharers’ coping and mental health (Orgilés et al., 2021), our study adopts the perspective of information receivers. We examine how viewers interpret and identify with sharers posting these two types of activities. This focus addresses the research gap by shifting the attention from sharers to viewers on the domain of personal brands.

Secondly, we show that perceived intrinsic motivation account for the social identification for posted activities. Existing research has predominantly focused on identity signaling and para-social interactions to explain how viewers make social identification with bloggers. For instance, consumers tend to identify with self-expressive products, as these products help communicate the sharer’s sense of self (Bellezza et al., 2014). Even the contents shared on social media can be shown as identity cues (Back et al., 2008). On the other hand, parasocial interaction induced by contents promotes consumer evaluations (Valsesia and Diehl, 2022; Zhang et al., 2024). Our research highlights the motivational inference process in social identification, showing that the posted behaviors shape consumers’ intrinsic motivation inferences, which in turn affects their social identification with personal brands.

Thirdly, this article identifies the societal and intrapersonal factors for the main effect. Prior research on self-improvement promotion primarily explores the varying effects of consumers’ personal traits and cultural contexts, such as emotional states (Allard and White, 2015), individual mindset (Du et al., 2025), religious beliefs (Grewal et al., 2022), and temporal perceptions. For example, individuals’ subjective perception of being busy can enhance their sense of pride, thereby increasing their preference for self-improvement products (Du et al., 2025). However, existing research lacks attention to some macro societal and interpersonal contextual factors. Our findings challenge the universal superiority of self-improvement by showing how social mobility and interpersonal similarity attenuate these benefits.

### 4.2 Managerial implications

First, this research offers actionable insights for social media influencers and content creators to consider disclosing self-improvement behaviors. For example, brands can regularly share contents related to self-growth, such as reading, learning and skill developing to cultivate a proactive and motivated brand image. Personal brands should strategically curate contents that clearly communicate an aspirational value such as sustainability, self-growth, and community-building. This fosters a strong in-group identity and builds a loyal following. In contrast, contents emphasizing on profit-driven motives, materialism, hedonic seeking or things related to extrinsic motivation pursuits may harm overall evaluations for influencers. So, it would be beneficial for hedonic bloggers to share hedonic activities along with some self-improvement activities.

Besides, influencers and online platforms should ramp up the self-improvement content during high social mobility periods. For example, brand advertising could focus on the appeal of self-improvement incentives and showcase their internal motivation for self-growth. Online platforms could invite celebrities or experts to share in-demand skills, such as artificial intelligence or data analysis, to encourage broader participation. When consumers perceive abundant opportunities for advancement, these efforts can help them connect with self-improvement communities more effectively. However, during periods of low social mobility periods, influencers and platforms should provide content in a more balanced way, encompassing both emotional wellbeing such as stress-relief tips and eudaimonic wellbeing such as personal fulfillment.

In addition, personal brands and firms should focus on creating personalized and emotionally engaging experiences to increase similarity between sharers and consumers. Since similarity promotes in-group recognition and alters perceptions of life goals, influencers and personal brands should tailor their shared content according to the similarity with brand communities. For example, when the similarity is high, brands have an opportunity to enhance the perceived intimacy and authenticity of their message through sharing hedonic activities. However, when perceived similarity is low, consumers need to clearly highlight their self-improvement efforts and showcase a positive image of internal life goals valuations. This might require a phased approach, where brands first build in-group attraction through self-improvement content, then gradually introduce their hedonic part of life when consumers become closer with the sharers in the brand community.

### 4.3 Limitations and future directions

Some limitations should be noted for future research. First, all experiments were conducted in China. Although the findings consistently support our research model, it would be valuable to examine whether the observed effects vary across Eastern and Western cultures. Different culture may vary in their masculinity aspirations and long-term orientations for self-improvement (Hofstede, 2016), which may influence their valuations different types of activities. Although we control for consumers’ general preference for self-improvement tendency, in more feminine societies such as Sweden and Norway, the positive effect of self-improvement may be attenuated. Second, this research examines both the impressions (Study 3) and engagements (Study 1, 2 and 4; liking, following, purchasing recommended products or engaging in promoted activities) for personal brand evaluation. Future research could explore more objective measures to replicate our effect, like prior online data from certain APP usage, content analysis via artificial intelligence, or consider real-life information sharing in a field experiment. Finally, we mainly distinguish between self-improvement and hedonic posts, overlooking the influence of other types of activities shared by bloggers. Although we ensure the realism of manipulation scenarios and further include a mixed condition (50% hedonic and 50% self-improvement posts), the combination ratio of the two behaviors may bring different results. Future research could take a continuity perspective and consider a broader range of activity sharing (Bogicevic et al., 2019), including social connection activities (i.e., family outings), responsibility-fulfillment activities (e.g., environmental protection), relaxation and recovery (e.g., meditation to recover energy).

### 4.4 Conclusion

Through four experimental studies, we demonstrate that sharing self-improvement posts improves personal brand evaluation compared to hedonic posts. Besides, such effect is driven by the perceived intrinsic motivational inferences for bloggers. Moreover, we also show that these effects would be mitigated in conditions with low social mobility or among viewers who feel a higher level of similarity to the bloggers.

## Data Availability

The raw data supporting the conclusions of this article will be made available by the authors, without undue reservation.

## Footnotes

^1^ www.credamo.com
