# Supplementary material for: Effort or ease: the impact of sharing self-improvement vs. hedonic behaviors on personal brand evaluation
Source: Front Psychol. 2025 Oct 20;16:1666105. doi: 10.3389/fpsyg.2025.1666105 (PMC12580192; doi:10.3389/fpsyg.2025.1666105)
Supplement: Supplementary file 1 [file Presentation_1.pdf]

Supplementary Material

Appendix A: *Manipulation Materials in Study 1, Study 2 and Study 4*

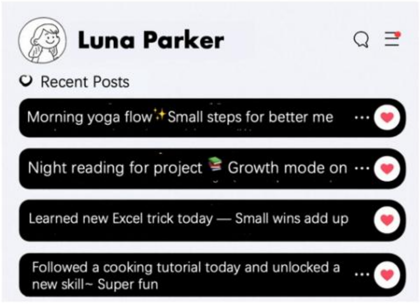

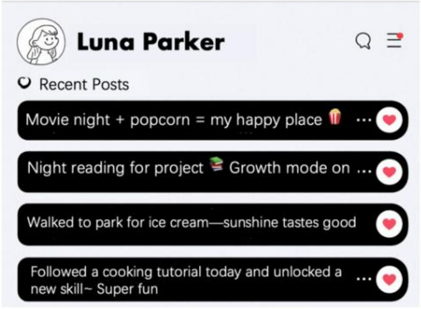

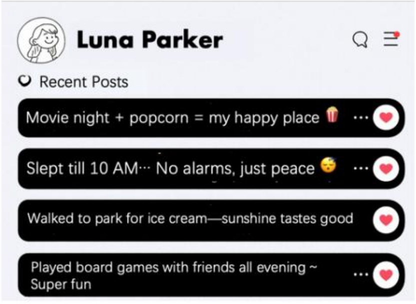

Self-improvement behaviors *Mixed behaviors* *Hedonic behaviors*

Appendix B: *Manipulation Materials in Study 3*

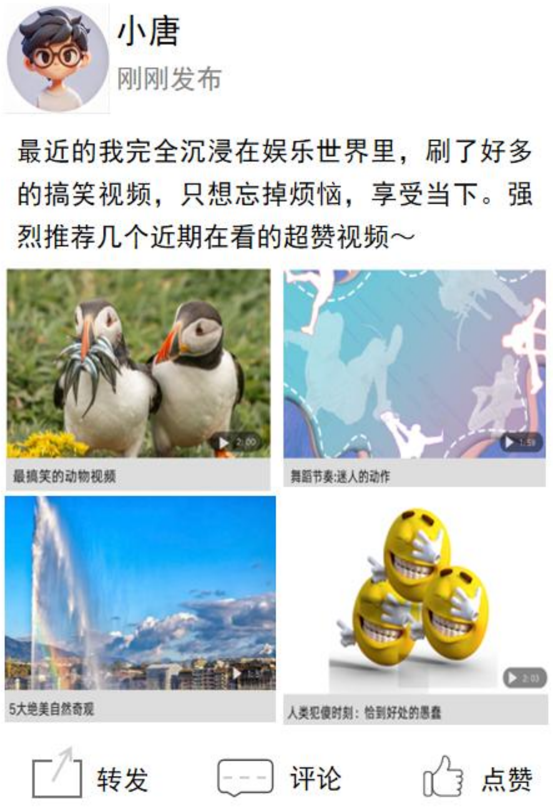

Hedonic behaviors

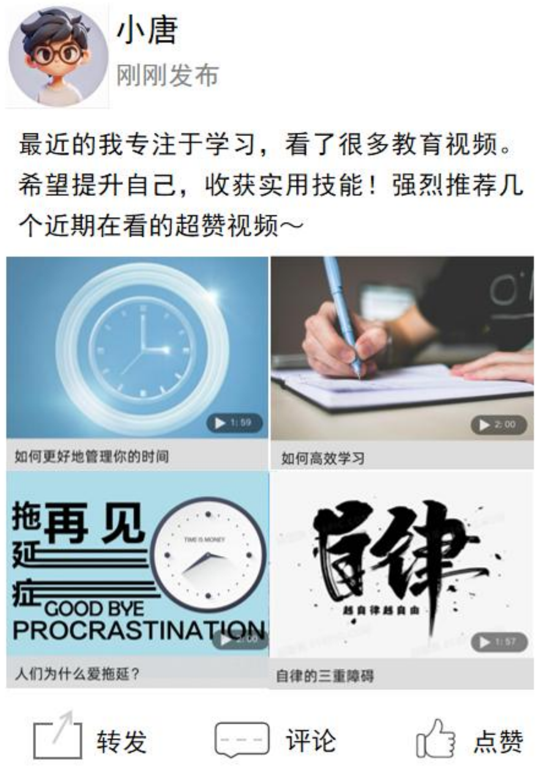

Self-improvement behaviors

Note: ***The English translation of the stimuli in the self-improvement condition*****:** Lately, I’ve been fully immersed in entertainment. I’ve watched all sorts of hilarious videos to just enjoy the moment and forget all my troubles. I’m excited to share with you some really great ones. ***The corresponding video titles***: Funniest Animal Videos; Dance Rhythms: Mesmerizing Moves; Breathtaking Natural Wonders; Humans Being Dumb: Perfectly Timed Stupidi.

The English translation of the stimuli in the hedonic condition: Lately, I’ve been deeply engaged in studying, watching various educational videos to enhance my skills and knowledge. I’m excited to share with you some really great ones. ***The corresponding video titles***: How to Manage Your Time Better; How to Study Efficiently; Why People Procrastinate; The Three Barriers to Self-Discipline.

The text in high social mobility condition:

Moving Towards Higher Ground

In 2025, a recent study analyzing combined cross-sectional data from the China General Social Survey (CGSS) and the China Labor-force Dynamics Survey (CLDS) reveals that individuals initially positioned in the bottom 20% of the social hierarchy have considerable opportunities for upward mobility. Although only about 5% of them reach the very top, approximately 66% manage to achieve higher incomes than their parents through their own efforts. In other words, many hardworking individuals from the lower strata eventually attain a higher social status than that of their parents.

Social mobility is not confined to the lower strata; those starting in the top 20% of the social spectrum may experience downward mobility as well. While only 6% of children from the highest social tier fall to the bottom layer in adulthood, about 40% earn significantly less than their parents, thus transitioning into lower income brackets.

Overall, children born into lower social strata can move upward, and conversely, some born at the top may not remain there. These upward and downward movements serve as important indicators of the health of social mobility. Although the current society may not be full of opportunities everywhere, the reality is that people possess numerous chances to change their social status. We are living in an era characterized by relatively high social mobility.

The text in low social mobility condition:

Moving to Higher Ground?

A recent 2025 study, analyzing combined cross-sectional data from the China General Social Survey (CGSS) and the China Labor-force Dynamics Survey (CLDS), finds that individuals initially positioned in the bottom 20% of the social hierarchy have only a 5% chance of advancing into the top 20% of social strata. In other words, many individuals starting at the bottom struggle to achieve upward mobility and are likely to remain in the same social class as their parents. Although some manage to make modest improvements relative to their parents, 66% of them fail to reach the middle-income range.

The issue of limited social mobility is not confined to the lower strata. Those whose incomes place them in the top 20% tend to remain there. Children born into affluent families have only a 6% likelihood of descending to the bottom social tier; most of them continue to earn in the highest or second-highest income brackets, similar to their parents.

Overall, children born into lower social strata are more likely to remain there, just as those born into the highest-income families tend to retain their elevated status. This lack of upward and downward movement indicates an unhealthy level of social mobility. Although there is a widespread hope for a society full of opportunities, the reality is that it is becoming increasingly difficult for individuals to change their social status. We are living in an era characterized by low social mobility.
